# Economic analyses to inform public health decision-making for tuberculosis: the role of understanding implementation

**DOI:** 10.1186/s12916-019-1468-5

**Published:** 2019-11-29

**Authors:** Priya B. Shete, James G. Kahn

**Affiliations:** 1 Division of Pulmonary and Critical Care Medicine, University of California San Francisco, Zuckerberg San Francisco General Hospital, 1001 Potrero Avenue, San Francisco, California 94110 USA; 2 Consortium to Assess Prevention Economics, Institute for Health Policy Studies, University of California San Francisco, 3333 California Avenue, San Francisco, California 94118 USA; 3 Department of Epidemiology and Biostatistics, University of California San Francisco, 550 16th Street, San Francisco, California 94158 USA

**Keywords:** Tuberculosis, Cost effectiveness, Public health system

## Background

Tuberculosis (TB) kills more people than any other infectious disease in the world today [1]. The global public health response is rising accordingly. To address the considerable gaps in TB management in high-burden countries, there is increasing pressure for health systems to rapidly and effectively implement innovations in TB diagnosis and treatment. High-level policy guidance, such as the World Health Organization’s End TB Strategy [2], the Sustainable Development Goals (SDG) [3], and the United Nation’s (UN’s) High Level Meeting in 2018 [4] all call for novel strategies to optimize the impact of these interventions. Modeling analyses, such as that recently published by Sohn and colleagues in *BMC Medicine*, are becoming increasingly instrumental in guiding policy decisions [5].

However, in most high-burden settings, it remains unknown how best to operationalize and scale up interventions, at a sustainable cost – critical concerns of stakeholders and decision-makers. Research exists, but it makes for poor generalizations. Delivery models for effective implementation of TB diagnosis and treatment innovations are often context-specific – influenced by local conditions in health system capacity and demand for services. Effectiveness is often narrowly or inconsistently defined. Together, these limitations greatly reduce the utility of cost-effectiveness and budgetary impact analyses, which are intended to inform the choices that programs must make about how to scale up novel technologies to maximize public health benefit.

## Cost drivers

In their paper, Sohn and colleagues consider these extensive planning needs. The authors meticulously conceptualize and implement an epidemiologic and economic model to demonstrate how a national public health system, such as India’s Revised National Tuberculosis Programme (RNTCP), can design a cost-effective deployment of rapid point-of-care molecular diagnostic tests for TB (GeneXpert, GeneXpert MTB/RIF Cepheid, Sunnyvale, USA) [5]. They describe three main drivers of cost-effectiveness for GeneXpert implementation: volume of testing, costs of sputum transportation in a decentralized approach, and the level of pre-treatment loss to follow-up for patients presenting to microscopy centers. These drivers are not unique to India; they have been described in a variety of high TB burden settings in both low- and middle-income countries [6, 7]. The strengths of this analysis are in its use of sensitivity and threshold analyses to characterize the factors that public health policymakers can consider or manipulate in designing an efficient approach to GeneXpert deployment. By applying context-specific cost and utilization assumptions, public health policymakers can reveal target conditions under which scale-up of GeneXpert would be cost-effective from a health system perspective (a major goal for economic analyses). But is this type of analysis sufficient, and how do policymakers know these costs for complex and under-used field activities?

Thus, challenges remain in comprehensive economic modeling of TB control. Drivers of GeneXpert cost-effectiveness, as described by Sohn and colleagues, notably depend on critical aspects of GeneXpert implementation and infrastructure. Human and capital costs related to infrastructure, capacity, and shared health systems resources, for example, are not routinely costed and, if mis-estimated, could skew results [8]. Empirical implementation costs that reflect supporting services or activities – either existing, or that would be required to achieve optimal effect – are left unacknowledged. Using the example of a decentralized approach to GeneXpert, this type of cost would include the true cost of starting, scaling and maintaining a sputum transportation network. From a previous cost-effectiveness analysis of GeneXpert implementation in India, Sohn and colleagues provide credible estimates for costs; however, they also acknowledge the limited empirical implementation-based cost data that may affect their results [5]. More broadly, economic evaluations also usually omit the perspectives of decision-makers such as patients, clinics, individual providers, and community organizations, for whom measures of effect are often different than traditional clinical efficacy. Cost drivers can also affect care participation, which will ultimately affect the fidelity of implementation and outcomes [9].

## Novel measure of effectiveness

It is a critical and continuing challenge to enhance cost and cost-effectiveness analyses to reflect real-world implementation and its constraints in TB. Many analyses would be more powerful for use in public health decision-making if they could realistically integrate costs for implementation strategies that optimize effectiveness. While they are a move in the right direction, studies using more real-world TB outcomes, such as case detection rates and pre-treatment loss to follow-up, are still the exception [8]. Additional outcomes of interest to stakeholders include those measuring integration, sustainability, and reach of novel interventions [10]. For example, while GeneXpert economic analyses acknowledge that decentralized testing services may become less cost-effective as volume of testing decreases and infrastructure costs increase, they neglect the positive impact that such decentralization may have on improving reach, accessibility, and equity of TB services for at-risk or underserved populations. Is it possible to quantify these less traditional outcomes and cost the intervention components necessary to achieve them in a way that is meaningful to decision-makers? Perhaps more importantly, is there a way to integrate a more expansive interpretation of ‘effectiveness’ in a way that allows decision-makers to choose implementation strategies for novel interventions that may be less efficient than the cost-effectiveness optimal, but ultimately more successful?

## Conclusions

In the future, innovative approaches to economic analyses that incorporate implementation factors will facilitate a more realistic and practical portrayal of the cost-effectiveness of TB innovations. This would provide a more actionable framework for public health decision-makers in programmatic and strategic planning to eliminate TB.

## Data Availability

Not applicable.
